# Decent Work: A Psychological Perspective

**DOI:** 10.3389/fpsyg.2016.00407

**Published:** 2016-03-24

**Authors:** David L. Blustein, Chad Olle, Alice Connors-Kellgren, A. J. Diamonti

**Affiliations:** Department of Counseling, Developmental, and Educational Psychology, Boston College, Chestnut HillMA, USA

**Keywords:** decent work, precarious work, Psychology-of-Working, career development, social justice and work

## Abstract

This contribution, which serves as the lead article for the Research Topic entitled “From Meaning of Working to Meaningful Lives: The Challenges of Expanding Decent Work,” explores current challenges in the development and operationalization of decent work. Based on an initiative from the International Labor Organization [ILO] (1999) decent work represents an aspirational statement about the quality of work that should be available to all people who seek to work around the globe. Within recent years, several critiques have been raised about decent work from various disciplines, highlighting concerns about a retreat from the social justice ethos that had initially defined the concept. In addition, other scholars have observed that decent work has not included a focus on the role of meaning and purpose at work. To address these concerns, we propose that a psychological perspective can help to revitalize the decent work agenda by infusing a more specific focus on individual experiences and by reconnecting decent work to its social justice origins. As an illustration of the advantages of a psychological perspective, we explore the rise of precarious work and also connect the decent work agenda to the Psychology-of-Working Framework and Theory (Blustein, 2006; Duffy et al., 2016).

## Introduction

The two specialties of industrial/organizational (I/O) and vocational psychology have been exploring work as a context for human experience and development for more than a century, producing substantive bodies of scholarship and practices that have positively impacted the lives of people, organizations, and communities (Savickas and Baker, 2005; Blustein, 2006; Guichard, 2009; Landy and Conte, 2010; Schleicher et al., 2011; Di Fabio and Maree, 2012; Di Fabio, 2014). At the present time, the labor market is undergoing radical transformations that are upending many of the taken-for-granted assumptions about work and careers (Blustein, 2013; Brynjolfsson and McAfee, 2014; Stiglitz, 2015). Working conditions are increasingly governed by market forces that are currently creating growing levels of instability and insecurity, thereby evoking greater levels of stress and anguish for people around the globe (Kalleberg, 2009; Paul and Moser, 2009; Richardson, 2012; Stiglitz, 2012, 2015; Guichard, 2013; Piketty, 2014; Standing, 2014). Amidst the radical changes in the world of work, international leaders from government, labor, and other public policy domains have provided needed guidelines about the quality of work that people should be able to access in contemporary society (International Labor Organization [ILO], 2008a,b, 2014, 2015; Standing, 2008). Their guidance has yielded an aspirational statement about the sort of work that ought to define the lives of all who work and who want to work—**decent work**.

As a means of understanding these changes and creating a knowledge base that will help to foster relevant research, we have developed this special Research Topic for *Frontiers in Psychology* entitled “From Meaning of Working to Meaningful Lives: The Challenges of Expanding Decent Work,” This article serves as an introduction to this special section and also provides a needed psychological examination of the concept of decent work, which is a key element of this project. When considered collectively, the articles that comprise this Research Topic build on the interface between I/O and vocational psychology (Carr et al., 2012; Blustein, 2013) by examining the complexities that people face as they seek to transition from school or unemployment to work and as they strive to adjust to an increasingly challenging workplace. In accordance with globalization trends and the intellectual pluralism that is defining the discourse on organizational and applied psychology (e.g., Di Fabio and Kenny, 2011; Carr et al., 2012; Blustein, 2013; MacLachlan, 2014), these contributions represent various settings around the world and also employ qualitative analyses, quantitative methods, case studies, and critical reviews, thereby furnishing readers with a broad canvas upon which to generate the next generation of scholarship to address these growing challenges to the world’s workforce. This introductory article provides a cohering thread, linking the articles by defining the psychological features of decent work and outlining its utility in enhancing the potential for people to create a life that includes fair, dignified, and decent work.

For the most part, policies, research, and advocacy on decent work have emerged from economics, public policy, sociology, governments, and the private sector. With the understanding that the availability of work that is stable and secure is associated with mental and physical health as well as greater cohesion in communities (Wilson, 1996; Paul and Moser, 2009; Swanson, 2012), we propose that psychologists need to understand and contribute to the conception of decent work, and join in ongoing dialogs about how to optimally create the conditions that promote decent work. To accomplish these goals, we seek to define decent work from the perspective of individuals and communities—in other words, what is the experience of decent work for people (as contrasted with the tradition of examining decent work from the perspective of macro-level markets) and what are the barriers that exist in attaining decent work?

While nuanced disagreements across disciplines remain as to what constitutes a good job, the conversation has progressed from subjective measures of job satisfaction to efforts by I/O psychologists (e.g., Hammer and Zimmerman, 2011) and vocational psychologists (e.g., Blustein, 2013; Lent and Brown, 2013), economists (e.g., Burchell et al., 2013), business management scholars (e.g., Vidal, 2013), and others (e.g., Deranty and MacMillan, 2012) to develop a consensus on the defining dimensions of high quality work. The use of a consensually agreed upon definition of decent work as fair, dignified, stable, and secure has the potential to drive research, policy initiatives, and potential solutions to the growing crisis in work.

While the International Labor Organization [ILO] (1999, 2008a,b) concept of decent work has been offered by some as an aspirational set of standards that can transform the nature of working, others have persuasively argued that there is a political battle for consensus within the ILO between stakeholders with competing interests (Standing, 2008; Deranty and MacMillan, 2012; Burchell et al., 2013; Di Ruggerio et al., 2015). In a thoughtful critical discourse analysis, Di Ruggerio et al. (2015) identified troubling themes in texts from the ILO, World Health Organization, and World Bank, suggesting a disconcerting shift of the ILO’s agenda away from social conceptualizations of work toward neoliberal, market-driven definitions following the global financial crash of 2008–2009. The move toward a more market-driven definition of decent work is a troubling development considering the historical influence of neoliberalism on governance, namely that the tendency of neoliberal social policy to privilege the individualization of work, health, and overall well-being (Rushton and Williams, 2012). Additionally, Piketty (2014) has presented painstakingly thorough evidence from 20 countries over three centuries that markets, left to their own devices, as is the goal of neoliberalism, will serve to elevate inequality. This political and ideological battle within the ILO effectively renders any consensus of aspirational standards a moving target, which, depending on who prevails, may or may not be consistent with a social justice and human rights agenda. The question becomes what can psychologists contribute to “redress(ing) the imbalance between economic and social framings of work to ensure that health and health equity remain at the forefront” of conceptualization of decent work (Di Ruggerio et al., 2015, p. 126)? In the next sections of this article, we explore a number of ways in which psychology can provide an important lens with which to understand the complexities and promise of decent work.

## Decent Work: An Overview

Decent work, as international concept, can be traced to the United Nations Declaration of Human Rights, which expressed the need for work as an integral aspect of human rights. The passage regarding work, which was endorsed by the General Assembly of the UN in December 1948, proposed the following:

(1) Everyone has the right to work, to free choice of employment, to just and favorable conditions of work and to protection against unemployment. (2) Everyone, without any discrimination, has the right to equal pay for equal work. (3) Everyone who works has the right to just and favorable remuneration ensuring for himself and his family an existence worthy of human dignity, and supplemented, if necessary, by other means of social protection. (4) Everyone has the right to form and to join trade unions for the protection of his interests (United Nations Declaration of Human Rights, 1948).

Building on this critical statement about work, the International Labor Organization (ILO), focused on defining the attributes of a work life that fulfills the mission of the UN Declaration. The International Labor Organization [ILO] (2008a, p. 6) advanced the notion of decent work as a statement to guide its agenda for the future of workers within an increasingly complex occupational context. The overarching objectives of the ILO Decent Work Agenda are “to meet the universal aspiration for social justice, to reach full employment, to ensure the sustainability of open societies and the global economy, to achieve social cohesion and to combat poverty and rising inequalities.”

The International Labor Organization’s [ILO] (2008a) definition of decent work, as stated in the report, includes the following attributes:

1. Promoting employment via sustainable institutional and economic contexts;
2. Defining, developing, and enriching social protection for workers, including social security and labor protection, which are constructed in accordance with the cultures of given societies;
3. Promoting social dialog via intentional connections among governments, worker organizations, and employers;
4. Affirming, advancing, and fulfilling the fundamental rights that define a dignified and just work place.

## Decent Work: Prevalence and Features

For the most part, decent work has been defined via macro-level economic factors, resulting in thoughtful analyses about the extent to which decent work is available in various countries and regions of the world (e.g., Ghai, 2003; Burchell et al., 2013; International Labor Organization [ILO], 2014). Much of the existing literature on decent work has used global indices of the labor market, including unemployment and underemployment rates, existence and proportion of child labor, employment-population ratio, conditions of work, availability of social security, access to basic rights at work, discrimination at work, freedom of association, and union density (Ghai, 2003; Standing, 2008). A perusal of the data in the report by Ghai (2003) and in more recent contributions from the International Labor Organization (ILO, 2014, 2015) reveals considerable variability with respect to access to decent work across the globe. Some countries, such as those from the Nordic region, do fare relatively well in these global indices of decent work (International Labor Organization [ILO], 2015). However, considerable gaps exist in the macro-level indices of decent work, which have become even more pronounced in recent years due to the major impacts of the Great Recession and the growing rise of automation (Guichard, 2013; Stiglitz, 2015). For example, the recent report by the International Labor Organization (ILO, 2015) reveals the unemployment rate will likely increase over the next 4 years by 8 million people globally. Similar views have been articulated by a panel of economic experts convened by the OECD Development Centre (2015), who have discussed some distressing developments in the world of work that echo the observations by the ILO.

As indicated by the trends identified by the International Labor Organization (ILO, 2015) and OECD Development Centre (2015) as well as scholars in economics (Burchell et al., 2013; Piketty, 2014) and psychology (Byars-Winston et al., 2012; Di Fabio, 2014), the crisis at the workplace is not likely to improve markedly in the coming years. The struggle to obtain decent work, particularly for workers without marketable 21st century skills, remains one of the major social justice challenges of our era. In this climate, we believe that linkages are needed between existing macro-level definitions of decent work and the psychological literature on quality of work life, social justice, and emancipatory views of human behavior (e.g., Prilleltensky, 1997; Blustein, 2006, 2013; Hammer and Zimmerman, 2011). In order to better understand the nature of the decent work concept, we first turn our attention to the emerging critiques, which have been generated from social philosophy (e.g., Deranty and MacMillan, 2012), economics (e.g., Burchell et al., 2013), public health (e.g., Di Ruggerio et al., 2015), and sociology (e.g., Standing, 2008). These critiques, in our view, provide the cohering thread between existing definitions of decent work and a psychologically infused conceptualization, which is the intention of this article and of this Research Topic.

## Decent Work: Critiques and a Way Forward

As indicated earlier, emerging concerns are being voiced about the ideological divisions present in the ILO’s tripartite governing structure, which includes representation from governments, the private sector, and workers. A similar set of concerns has been articulated by Standing (2008), who was involved in the development of the International Labor Organization [ILO] (1999) position paper about decent work. Standing’s critique centered around some of the same issues that Di Ruggerio et al. (2015) described, notably the relative retreat from a more assertive position with respect to workers’ rights. He indicated that due to organizational problems within the ILO and the diffusion of an explicitly justice-oriented agenda, existing definitions of decent work are replete with vagueness and ambiguity.

In addition to the concerns about the dominating influence of market-based forces, another theme has emerged that pertains to the relative neglect of psychological notions of work within existing views of decent work. This position is best articulated in the social philosophical critique by Deranty and MacMillan (2012), who have argued that internal constructions of meaning at work are excluded from existing formulations of decent work. Informed by the psychodynamic theory of working developed by Dejours (2006), Deranty and MacMillan (2012) constructed a compelling argument that decent work needs to include the perspectives of working people themselves. They proposed that decent work also needs to be meaningful work, which is a position that parallels many existing formulations in psychology about meaning at work (e.g., Savickas, 2011; Dik et al., 2013).

Legal concerns also have been raised about the decent work agenda. A defining feature of the decent work agenda has been its aspirational nature; in effect, the proposals to advance decent work are generally not based on legal mandates or policies that have been endorsed by governments (Deranty and MacMillan, 2012). A thorough analysis of the legal complexities inherent in advocating for decent work has been provided by MacNaughton and Frey (2011). These legal scholars provided a compelling rationale for using a holistic human rights framework to help establish the legal context for the decent work agenda. That said, their article identified the complexity in moving from an aspirational set of principles to legal structures that can result in systemic change in people’s experiences with work.

When considered collectively, the critiques that we have reviewed herein point to a growing lack of consensus with respect to the values that are integral to notions of decent work. Rather than the clear posture of the earliest developers of decent work which explicitly endorsed a human rights view characterized by a strong rejection of prevailing views of working people as economic commodities (International Labor Organization [ILO], 1999), the current trend seems to be increasingly responding to market forces (cf. Di Ruggerio et al., 2015). While we have argued that the macro-level perspectives used in original conceptualizations are clearly welcome in psychology to provide external criteria with which to evaluate work-based policies, we believe that a bridge needs to be created between these macro-level perspectives, which are increasingly vulnerable to outside influences from employers and neo-liberal policies, and the lived experience of working people. In our view, this bridge can be constructed via the scaffolding of psychological theory and research about work. Additional support for this bridge needs to come from the critical perspectives that have been reviewed previously, which are conceptually connected to the social justice-based ideas that are increasingly emerging in psychological discourse about work and careers (e.g., Richardson, 1993, 2012; Blustein, 2006, 2013; Carr et al., 2012; Flores, 2013).

In the sections that follow, we first review the relationship between work and psychological health, which underscores the advantages of adopting a psychological perspective of decent work. In order to provide an exemplar of the utility of a psychological lens in the decent work discourse, we explore the emergence of precarious work and its impact on workers, particularly their health and well-being. Then, from a Psychology of Working Framework (PWF; Blustein, 2006), we will position a socially constructed aspiration of decent work for all who would like stable, dignified, and secure work as the antidote to precarious work. We propose that the PWF can function as a needed conceptual framework for the decent work agenda. In effect, our view is that the concept of decent work can enhance the psychological study of work and careers, and that a psychological standpoint can enrich the decent work agenda. This sort of intentional synthesis may help to integrate the diverse streams of scholarship on unemployment, precarious work, social oppression, and other forms of “bad work” environments that continue to plague the labor market. Finally, we discuss how the integration of decent work and PWF will promote a needed synthesis of research and public policy on workplace initiatives and labor policy, which has tended to neglect the important contributions from psychology.

## Work and Psychological Health

Considerable research within psychology has detailed the various ways that access to work promotes psychological health (Blustein, 2008; Swanson, 2012). In this section, we examine this literature with a particular focus on how the decent elements of work may be critical in understanding the relationship between work and psychological health. As detailed in an excellent review by Swanson (2012), extensive research exists that supports the basic premise that working is associated with psychological health and well-being. In this article, we define psychological health as encompassing not simply the absence of mental health problems (cf. Swanson, 2012), but in accordance with the World Health Organization (WHO), “as a state of well-being in which every individual realizes his or her own potential, can cope with the normal stresses of life, can work productively and fruitfully, and is able to make a contribution to her or his community” (World Health Organization [WHO], 2014).

The Swanson (2012) contribution explored two specific lines of research that explicated the relationship between work and psychological health. First, Swanson (2012) described the extensive scholarship that has identified a significant and pernicious rise in mental health problems for individuals who are unemployed for 6 months for more (see Paul and Moser, 2009, for a detailed meta-analysis on this issue). She also reported research that documented improvements in mental health once people became reemployed (cf. Paul and Moser, 2009; Wanberg, 2012). Second, Swanson (2012) reviewed research that has detailed the various ways that work supports psychological health, such as promoting an adaptive family-work balance and enhancing various indices of adaptive well-being (such as quality of life, life satisfaction, etc.). However, as recent research from Australia has indicated (e.g., Butterworth et al., 2013), not all jobs are associated with gains in psychological health. Many jobs present people with psychologically and physically painful experiences, exposure to various forms of social oppression and marginalization, boredom, exhaustion, and other sources of physical and psychic distress (Blustein, 2006).

## The Increasing Prevalence of Precarious Work

One of the most important contributions of the decent work concept and agenda is the acknowledgment that by ignoring the quality of work available, conventional indicators like unemployment statistics reveal little about how well a labor market is meeting the needs of a society and its workers. Likewise, an emerging literature in the social sciences is highlighting growing concerns about rising levels of precarious work. Although a full consensus has not been reached among scholars regarding its definition, precarious work is generally understood as a multidimensional construct defined along four dimensions: continuity/employment insecurity, vulnerability (i.e., powerlessness/lack of bargaining position or ability to exercise workplace rights), protection (i.e., access to benefits and legal protections), and income (Benach et al., 2014). Precarious workers typically lack effective agency and have little bargaining power, or means of resisting exploitative and oppressive labor conditions, leaving them little choice other than to abide by market forces or face severe consequences of not being able to find work and maintain their livelihoods (Standing, 2014).

Although precarity has been a continuous feature of work in developing nations, its prevalence in Western economies had declined during the 20th century, as expansions of social protections, regulation of workplace conditions, the promotion of collective bargaining, and the rise of union representation successfully addressed many of the problems, and reduced the occurrence of precarious employment (Menéndez et al., 2007; Evans and Gibb, 2009). However, since the 1970s, employment relationships have again been undergoing substantial changes, this time emerging from neoliberal restructuring of labor markets as governments and business have sought to respond to the effects of globalization, growing global competition, rapid technological advances, and a changing labor force (Merolli, 2012). In accordance with a neoliberal agenda, which emphasizes the effectiveness of markets to self-regulate and respond efficiently to change, governments have increasingly adopted policies aimed at deregulation, and supportive of corporate *‘flexibility*,*’* often in ways that have eroded employment standards and shifted social risk away from businesses, with adverse effects on workers (Evans and Gibb, 2009). Among the consequences of policies aimed at promoting corporate flexibility, growing precarious employment shifts risks away from employers (and governments) and places them instead on workers, their families, and communities. As a result, the burden of risk is now being shouldered by those who are least able to bear it (Evans and Gibb, 2009). Modern precarity, unlike its pre-WWII incarnation, is notable because it has spread to all sectors of the economy, including occupations that were historically seen as secure or permanent (Malenfant et al., 2007; Kalleberg, 2008; Facey and Eakin, 2010; Quinlan, 2012). Many workers are now facing job insecurity not as a transient condition on the path to permanent employment, or a temporary setback, but as a chronic situation in their lives, and consequently, an ongoing source of stress (Artazcoz et al., 2005; Lipscomb et al., 2006). Increased occupational stress, sustained uncertainty due to the threat of job loss, and a lack of control over the future, leads many workers to overwork and/or avoid taking needed time off in order to maintain employment (Clarke et al., 2007; Malenfant et al., 2007).

Not surprisingly, a growing body of research supports the conclusion that precarious employment has deleterious health effects (Lewchuck et al., 2003; Facey and Eakin, 2010). Multiple studies have found a negative impact of casual or intermittent work, and related experiences of insecurity, on well-being, self-esteem, and social recognition, all of which were as damaging to workers’ mental health as the stress and insecurities linked to unemployment (e.g., Artazcoz et al., 2005; Malenfant et al., 2007). Workers experiencing chronic job insecurity had the highest morbidity on self-report measures, made more frequent use of health services, and had higher rates of adverse physiological indicators and cardiovascular risk factors (e.g., high blood pressure, increased serum cortisol, increased Body Mass Index ratios; Lipscomb et al., 2006; Benach and Muntaner, 2007). Perceived job insecurity associated with precarious employment has also been linked to increased prevalence of depressive symptoms and generalized anxiety in a preponderance of studies examining the issue (Benach et al., 2012, 2014). As a result, precarious employment has come to be considered a social determinant of health, with well-documented aversive effects on workers, families, and communities (Benach et al., 2014).

Although workers in these circumstances are still technically employed, precarious employment involves a loss of many of the latent functions of work, such as the development of a sense of adult identity, a sense of purpose, and inclusion in social organizations (Blustein, 2006; Benach et al., 2014). Thus, notable among the factors identified in empirical studies as leading to negative health outcomes are a lack of recognition (most often characterized by low pay and lack of respect from colleagues), job insecurity, restricted autonomy, limited possibility for advancement or to develop one’s abilities, lack of work support, and the intensification of work (Malenfant et al., 2007; Benach et al., 2014). Precarious workers also typically gain fewer social connections through work, and experience a comparative lack of social support (Clarke et al., 2007; Evans and Gibb, 2009) along with increased social isolation, which are known psychological stressors (Blustein, 2006, 2011; Swanson, 2012; Blustein et al., 2013; Flum, 2015).

Considered collectively, this evidence suggests that the impact of employment on health depends more on the quality of work than simply the obtainment of work. We would further make the case that precarious work and unemployment actually occur along a continuum. In fact, expanding this continuum to include the adaptive concept of decent work will help to provide locations for indexing the complexity and diversity of contemporary work experiences and will provide scholars with a means of understanding the various ways that work can provide meaning and purpose, as well as stability and economic security.

## Moderating Factors

Although precarious employment has been associated with increased physical health risks in the workplace, Clarke et al. (2007) suggest that the relational characteristics of employment and the changing nature of the social structure of employment better explain the social/psychological health outcomes of precarious employment. Whether individuals want more permanent employment, and whether they believe this goal is attainable, may be central in understanding the impact of precarious employment on health and mental health. For example, the small population that does seem to thrive under conditions of precarious employment seems to have access to greater collective and individual sources of support (Kalleberg, 2008). Workers who engage in temporary work “voluntarily” are more likely to have resources enabling them to seek out employment arrangements that enhance their quality of life, whereas employees who accept temporary work “involuntarily” are significantly more likely to experience job dissatisfaction and stress (Benach and Muntaner, 2007).

Often, these individuals have greater access to healthcare benefits, a partner who is stably employed with adequate earnings, and workplace supports (such as training and opportunities for social networking), all of which have been found to mitigate the impact of workplace precarity (Clarke et al., 2007). Additionally, several studies (e.g., Artazcoz et al., 2004; Clarke et al., 2007) have found evidence that social and systemic support were essential to the health of precarious workers, and that negative mental health effects were particularly associated with precarious work among less educated workers, women, and ethnic minorities. Thus those who most need support are arguably among the least likely to receive it.

Indeed, social marginalization plays a substantial role in determining who has access to decent work (Ali, 2013; Flores, 2013; Duffy et al., 2016). Employment conditions, such as precarious, insecure or low-paying jobs, child labor, and work in hazardous conditions, significantly influence individual, family, and community health and thereby, inequality. Such employment conditions have a notably differential impact on health across social classes, racial or ethnic groups, and gender (Quinlan, 2012). Access to decent work provides a well-established pathway out of poverty and marginalization (Duffy et al., 2016). Yet, as Lipscomb et al. (2006) note, the nature and quality of benefits afforded by work vary by class, race, and gender in ways that affect health, and contribute to disparities among groups.

In sum, the literature on precarity provides an informative exemplar of how a psychological perspective of decent work can help to illuminate issues regarding the quality of a given job. The rise of precarious work underscores the need for a comprehensive definition of decent work that explicitly captures the psychological aspects of working. As reflected in the next section, a psychological explication of decent work, based on the PWF, has the potential to further clarify the definitional boundaries and contours of the concept.

## The Psychology of Decent Work

Although the decent work agenda has not been formally integrated within psychology, considerable psychological theory and research has focused on various aspects of work that correspond with existing definitions of decent work. For example, substantial research has been devoted to identifying the nature and predictors of people finding a good fit in their work lives; various indices exist within vocational and I/O psychology to assess the extent to which a given job fits the values, interests, attitudes, and abilities of a given worker (see for example, Holland, 1997; Dawis, 2005; Schleicher et al., 2011). In vocational psychology, extensive effort has been devoted to understanding and predicting the nature of a good person-environment fit, as reflected in the seminal theory development efforts by Holland (1997) and Dawis (2005). Empirical research within vocational psychology has documented that individuals whose work environments fit well with their interests, values, and abilities are more likely to experience job satisfaction (Nauta, 2013) and other indices of well-being at work (Lent and Brown, 2013). Moreover, individuals who have the opportunity to experience job satisfaction also are more likely to have other forms of well-being and psychological health in their lives (Swanson, 2012; Lent and Brown, 2013). In addition, psychologists from the I/O and work psychology traditions have explored the various ways that people can obtain quality of life at work (Hammer and Zimmerman, 2011), job satisfaction (Schleicher et al., 2011), and other forms of meaning at work. Indeed, the scholarship within vocational and I/O psychology has generated considerable relevant knowledge in understanding many of the psychological attributes of a functional work environment (see Lent and Brown, 2013, for an excellent review of the existing research in both I/O and vocational psychology).

## A Psychology of Working Perspective on Decent Work

The PWF (Blustein, 2006, 2008) was initially advanced as a critique of existing discourses in vocational psychology that had privileged the lives of people who had some individual control over their career choices. As the critique was fully developed, a meta-perspective was constructed that provided a rich exploration of the psychological nature of contemporary working experiences (Blustein, 2006, 2013). Consistent with the decent work agenda, the PWF incorporates an activist, social justice perspective that seeks to link individual analyses of work-related issues to broader social and economic factors, which clearly play a key role in understanding the distribution of resources and access to decent work (Blustein, 2006, 2013). More recently, Duffy et al. (2016) have constructed a precise theoretical statement, known as the Psychology-of-Working Theory (PWT), which has sought to identify the salient antecedents and consequences of decent work. The PWT posits an empirically testable model based on the concepts outlined in the PWF that places decent work at the center of work experiences for all individuals. This theoretical model includes psychological factors such as proactive personality, career adaptability, and critical consciousness along with social and economic factors, such as economic conditions, marginalization, and social class. These two contributions seek to understand the diverse work experiences of individuals from different backgrounds, particularly those belonging to marginalized and disenfranchised social groups who have historically had less access to traditional career narratives, a group that is growing as work becomes increasingly precarious (Standing, 2008; International Labor Organization [ILO], 2015). Furthermore, the PWF and PWT pay attention to the ways in which sociocultural factors, such as discrimination, oppression, intersectional identities, high barriers, and low volition, affect the career development process and experience of work. By expanding the definition of work and those who engage in the world of work to include every person who is involved in market or care work, the Psychology-of-Working movement advances a view of work as a human right central to mental health and wellbeing through its ability to meet three basic needs: survival and power, social connection, and self-determination (Blustein, 2006, 2013; Duffy et al., 2016). Due to the variations in the nature of different jobs, these needs are additive and are not mutually exclusive, meaning that people can achieve wellbeing through different combinations and levels of fulfillment with each need, that multiple needs may be met by the same facet of work, and that gratification of one need may bolster the fulfillment of other needs.

Decent work, as outlined by the International Labor Organization [ILO] (2008a), has the inherent capacity to meet the three needs set forth by the PWF. Because the needs outlined by the PWF can be fulfilled to varying degrees and in varying arrangements, the PWF, like decent work, provides an aspirational frame for work. To further provide a more structured framework to the integrated vision of decent work, as well as to promote the psychological aspects essential to truly defining decent work, we will outline the ways in which it provides or interacts with the needs posited by the PWF.

Survival and power, as posited by the PWF (Blustein, 2006), comprise one need defined as an individual’s access to work that ensures survival and the capacity to make his or her objectives prevail. Survival is fulfilled by work characteristics such as job security, job stability, provision of a living wage, benefits such as health insurance and paid time off, and a sense of independence and control in the work place. Each of these characteristics can also be considered as part of the definition of a decent work environment. Characteristics of work that fulfill survival needs exist at the workplace level, as well as at the policy and macroeconomic levels. The extent to which individuals’ survival needs are met by work can serve as one important dimension of decent work in individual jobs or workplaces and can be scaled to assess how well countries are meeting the aspiration of decent work. For example, although individual workplaces may vary in the wages and benefits they offer employees, governments have the ability to set minimum wages and offer safety nets to their citizens that reduce the negative consequences of precarious work.

The second need outlined by the PWF is social connection, which describes the aspects of work that provide access to relationships with others, as well as a sense of connection to society and the world at large (Blustein, 2006, 2011; Flum, 2015). Work can fulfill this need through a supportive, respectful environment – regardless of an individual’s identity or social location – and through policies that provide time and resources for individuals to maintain positive relationships outside of work (e.g., paid family leave). Like the facets of work that fulfill the survival need, the characteristics of work that lead to social connection can be influenced at multiple levels connected to employment.

Finally, the PWF asserts that work can and should fulfill the need that humans have for self-determination, or the development of meaning for jobs, whether or not they are inherently intrinsically rewarding (Blustein, 2006). Self-determination in the workplace is generated by opportunities for autonomy, relatedness, and competence, with an emphasis on value congruence and access to the opportunity structure (Blustein, 2006). When individuals have the ability to locate and make use of resources and supports that bolster successful work experiences within their jobs, fields, or the world of work writ large, they are more likely to develop a sense of self-determination in the realm of work (Duffy et al., 2016). In terms of decent work, employers and governments should aspire to increase the volition that individuals have in choosing work through easy access to quality education, increased training and vocational programs, and equal access to opportunities for independence and advancement within employment organizations. Psychologically, the ability to gain a sense of satisfaction, autonomy, and competence from one’s work is integral to the universal vision of decent work (Deranty and MacMillan, 2012).

By working toward the fulfillment of survival, social connection, and self-determination needs as outlined by the PWF, the decent work agenda can gain clarity and form while placing an emphasis on the psychological health and wellbeing of workers around the world. To further add specificity and depth to this structure, scholars, policymakers, and human rights advocates can draw on the emerging PWT (Duffy et al., 2016), which represents the next logical step within the PWF movement. Research stemming from the PWT can lead to recommendations about the specific stepping stones needed to reach the goal of decent work for all, as well as a model for evaluating the extent to which employers and governments have met the integrated vision of decent work across the globe.

## Psychology and Decent Work: Concluding Points

Employment conditions are determined by a combination of labor markets and social policy (Quinlan, 2012). Given the dominance of neoliberal ideology and agendas and the rise in automation (Piketty, 2014; Stiglitz, 2015), the current growth in precarious employment is not likely to diminish any time soon. In this context, the psychological health consequences of precarious or non-standard employment conditions remain a neglected concern, particularly as such work-related inequalities remain somewhat invisible in economically prosperous countries (Benach et al., 2010). One of the directions that is suggested by our discussion is the use of qualitative, discovery-oriented research as a tool to unpack how people experience their working contexts. The use of rigorous and relevant narrative data on the nature of working may help to respond to some of the critiques of the decent work agenda (Burchell et al., 2013; Di Ruggerio et al., 2015), which has been so heavily rooted in statistics and macro-level data, thereby missing important aspects of people’s lived experiences at work. In our view, qualitative research may be particularly informative in detailing the impact of the growth in precarious work.

Consistent with Deranty and MacMillan’s (2012) thoughtful critique of decent work, we have sought to provide an initial map of an integrative landscape that embraces both psychology and decent work. As reflected in the material that we have reviewed, the growth in precarious work is evoking greater problems for people in maintaining their sense of stability, health, and well-being at work. The infusion of the PWF provides a meta-perspective that may help to delineate the complex ways that working fulfills core human needs. Moreover, the socio-political context of the PWF and the new PWT (Duffy et al., 2016) parallel the focus on the importance of creating macro-level conditions that will nurture our inherent need to contribute, collaborate, and create. In particular, research derived from the PWT can provide an integrative rubric for considering the complexity of how macro-level factors, such as social and economic conditions, interface with individual psychological experiences. Indeed, one of the major recommendations that we advance here is that psychologists develop collaborative research groups with other social scientists and policy makers to examine the psychological and social antecedents and consequences of decent work. The PWT is one viable tool to stimulate this research; the articles in this Research Topic coupled with the input from the thoughtful critics of decent work can also inform much needed scholarship that can shape policy.

In keeping with the decent work agenda that prioritizes health and health equity, we position political and economic systems of organizations, that is governments and economies, as the next frontier for psychologists interested in work and careers—the next unit of analysis and deconstruction on the basis of how well they facilitate equitable access to decent work. Many psychologists recognize that the environment and individual recursively influence each other; however, by not engaging directly in political and economic discourse, they may inadvertently endorse structures and ideologies that are deterministic, ahistorical, and serve to over-individualize the responsibility for psychological health (Rushton and Williams, 2012). A research and policy agenda infused with the psychology of working will protect us from being unaware of or complacent in our tacit endorsements of systems whose logic and ideology runs counter to our stated values, assumptions, and practices as psychologists concerned with social justice (Prilleltensky, 1997).

As psychological research-practitioners, we recognize that the integrity of our inquiry, analysis, and interpretation is vital to maintaining trust in psychology as a discipline concerned with advancing the public good. However, contrary to Burchell et al. (2013), we find it unlikely that even the most rigorously researched scientific findings will be privileged above the fray of clashing political and economic interests merely because they are less overtly political. We argue that psychologists should not shy away from being political when human health and health equity, our foundational ethos, are thrust into the political arena by conflicting interests. An adoption of a decent work agenda grounded in the social justice principles that inspired this movement means not only using the theory and research that illuminated the conditions of a just society to advocate for those conditions, but using psychology to be more aware of political interests that threaten their realization. Our hope is that the psychological perspective advanced in this article, coupled with the other contributions in this Research Topic, will serve to revitalize the decent work agenda so that it can clearly and forcefully set standards for work that is safe, secure, meaningful dignified, and consistent with the best aspects of the human spirit.

## Author Contributions

DB organized the project, created the basic framework for the paper, and wrote a significant portion of the manuscript. AC-K, CO, and AD worked closely with DB in the construction of the outline and in suggesting various bodies of work to include in the manuscript. Each of the co-authors wrote substantial sections of the paper and each edited the entire manuscript multiple times prior to submission.

## Conflict of Interest Statement

The authors declare that the research was conducted in the absence of any commercial or financial relationships that could be construed as a potential conflict of interest.
